# Geographic disparities and predictors of COVID-19 vaccination in Missouri: a retrospective ecological study

**DOI:** 10.3389/fpubh.2024.1329382

**Published:** 2024-03-11

**Authors:** Alexanderia Lacy, Md Marufuzzaman Khan, Nirmalendu Deb Nath, Praachi Das, Morganne Igoe, Suzanne Lenhart, Alun L. Lloyd, Cristina Lanzas, Agricola Odoi

**Affiliations:** ^1^Department of Mathematics, University of Tennessee, Knoxville, TN, United States; ^2^Department of Public Health, University of Tennessee, Knoxville, TN, United States; ^3^Department of Biomedical and Diagnostics Sciences, University of Tennessee, Knoxville, TN, United States; ^4^Biomathematics Graduate Program, North Carolina State University, Raleigh, NC, United States; ^5^Department of Population Health and Pathobiology and Comparative Medicine Institute, North Carolina State University, Raleigh, NC, United States

**Keywords:** COVID-19, Missouri, spatial epidemiology, epidemiology, geographic disparities, predictors, vaccination

## Abstract

**Background:**

Limited information is available on geographic disparities of COVID-19 vaccination in Missouri and yet this information is essential for guiding efforts to improve vaccination coverage. Therefore, the objectives of this study were to (a) investigate geographic disparities in the proportion of the population vaccinated against COVID-19 in Missouri and (b) identify socioeconomic and demographic predictors of the identified disparities.

**Methods:**

The COVID-19 vaccination data for time period January 1 to December 31, 2021 were obtained from the Missouri Department of Health. County-level data on socioeconomic and demographic factors were downloaded from the 2020 American Community Survey. Proportions of county population vaccinated against COVID-19 were computed and displayed on choropleth maps. Global ordinary least square regression model and local geographically weighted regression model were used to identify predictors of proportions of COVID-19 vaccinated population.

**Results:**

Counties located in eastern Missouri tended to have high proportions of COVID-19 vaccinated population while low proportions were observed in the southernmost part of the state. Counties with low proportions of population vaccinated against COVID-19 tended to have high percentages of Hispanic/Latino population (*p* = 0.046), individuals living below the poverty level (*p* = 0.049), and uninsured (*p* = 0.015) populations. The strength of association between proportion of COVID-19 vaccinated population and percentage of Hispanic/Latino population varied by geographic location.

**Conclusion:**

The study findings confirm geographic disparities of proportions of COVID-19 vaccinated population in Missouri. Study findings are useful for guiding programs geared at improving vaccination coverage and uptake by targeting resources to areas with low proportions of vaccinated individuals.

## 1 Background

Coronavirus Disease 2019 (COVID-19) is a highly infectious disease caused by the Severe Acute Respiratory Syndrome Coronavirus 2 (SARS-CoV-2). The first COVID-19 case was identified in Wuhan, China, in December 2019 (1), and it was declared a pandemic by the World Health Organization (WHO) the following year. As of March 2023, there have been more than 103 million confirmed cases and over 1.1 million deaths in the United States (US) (2). The state of Missouri detected the first confirmed case on March 8, 2020 (3), and has reported more than 1.7 million COVID-19 cases and 22 thousand deaths as of March 10, 2023 (4).

Vaccination is an effective way to reduce the risk of COVID-19 infections. The US Food and Drug Administration (FDA) authorized the emergency use of COVID-19 vaccines on December 2020 (5), and since then, vaccines have been administered all over the US. However, evidence shows that the COVID-19 burden and vaccine uptake vary geographically due to sociodemographic factors and population characteristics, as well as inequities in healthcare accessibility among populations (6–9). According to the Centers for Disease Control and Prevention (CDC), non-Hispanic Black and Hispanic populations have a higher risk of COVID-19, and yet they are less likely to be vaccinated compared to non-Hispanic White and populations of other racial categories (10, 11). Evidence also suggests that educational attainment, poverty, occupation, rurality, and healthcare access are associated with COVID-19 vaccine hesitancy and vaccine coverage (12–14). Additionally, concerns over misinformation and speed of vaccine development have impacted vaccine acceptance, as highlighted by public health experts and the WHO (15–17).

As of September 2022, the state of Missouri fully vaccinated only 58.9% of the total population (18, 19), which fell far behind the national average (70%) and ranked Missouri as the 11th lowest vaccinated state in the US. In addition, the findings of a recent study conducted among undergraduate students of a university in Missouri reported that several socioeconomic and demographic factors, such as access to healthcare facilities, availability of primary care physicians, and health insurance, were associated with vaccine hesitancy (19). Evidence suggests that vaccine hesitancy is a major barrier of vaccination coverage (20, 21). However, very little is known about the geographic disparities and predictors of COVID-19 vaccination in Missouri. This knowledge is essential for identifying communities with low COVID-19 vaccination in Missouri and guiding targeted planning to improve vaccination coverage in the state. Therefore, the objective of this study was to investigate county-level geographic disparities and predictors of COVID-19 vaccination in Missouri.

## 2 Materials and methods

### 2.1 Ethics approval

Ethical review and approval was not required for the current study in accordance with the local legislation and institutional requirements.

### 2.2 Study design and area

**Figure 1:**
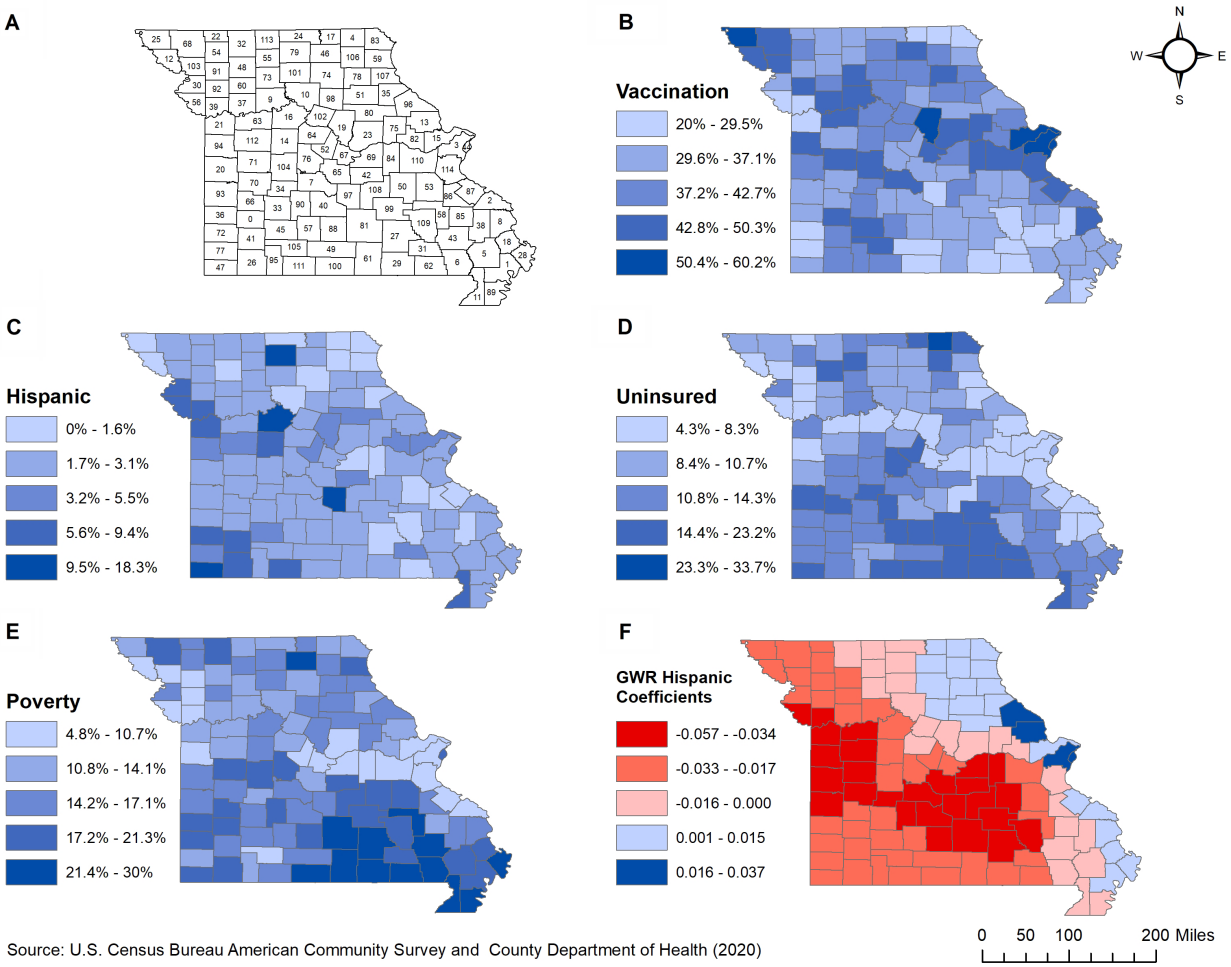
Geographic distribution of: **(A)** counties, **(B)** proportions of COVID-19 vaccinated population, **(C–E)** significant predictors, and **(F)** spatially varying local coefficients in Missouri. 0, Dade; 1, New Madrid; 2, Perry; 3, St. Louis; 4, Scotland; 5, Stoddard; 6, Butler; 7, Camden; 8, Cape Girardeau; 9, Carroll; 10, Chariton; 11, Dunklin; 12, Holt; 13, Lincoln; 14, Pettis; 15, St. Charles; 16, Saline; 17, Schuyler; 18, Scott; 19, Boone; 20, Bates; 21, Jackson; 22, Worth; 23, Callaway; 24, Putnam; 25, Atchison; 26, Barry; 27, Shannon; 28, Mississippi; 29, Oregon; 30, Buchanan; 31, Carter; 32, Harrison; 33, Polk; 34, Hickory; 35, Ralls; 36, Barton; 37, Ray; 38, Bollinger; 39, Clay; 40, Laclede; 41, Lawrence; 42, Maries; 43, Wayne; 44, St. Louis; 45, Greene; 46, Adair; 47, McDonald; 48, Daviess; 49, Douglas; 50, Crawford; 51, Monroe; 52, Moniteau; 53, Washington; 54, Gentry; 55, Grundy; 56, Platte; 57, Webster; 58, Iron; 59, Lewis; 60, Caldwell; 61, Howell; 62, Ripley; 63, Lafayette; 64, Cooper; 65, Miller; 66, Cedar; 67, Cole; 68, Nodaway; 69, Osage; 70, St. Clair; 71, Henry; 72, Jasper; 73, Livingston; 74, Macon; 75, Montgomery; 76, Morgan; 77, Newton; 78, Shelby; 79, Sullivan; 80, Audrain; 81, Texas; 82, Warren; 83, Clark; 84, Gasconade; 85, Madison; 86, St. Francois; 87, Ste. Genevieve; 88, Wright; 89, Pemiscot; 90, Dallas; 91, DeKalb; 92, Clinton; 93, Vernon; 94, Cass; 95, Stone; 96, Pike; 97, Pulaski; 98, Randolph; 99, Dent; 100, Ozark; 101, Linn; 102, Howard; 103, Andrew; 104, Benton; 105, Christian; 106, Knox; 107, Marion; 108, Phelps; 109, Reynolds; 110, Franklin; 111, Taney; 112, Johnson; 113, Mercer; 114, Jefferson.

This retrospective ecological study was conducted in 2022–2023 in the state of Missouri, which consists of 114 counties (Figure 1A). Missouri has a population of approximately 6 million, with 50.6% female and 49.4% male residents. Most of the residents are White (82.6%). Black or African American comprise 11.8% of the population while the rest (5.6%) are from other categories that include American Indian, Alaska Native, Asian, Native Hawaiian, Other Pacific Islander, and multiracial groups (22). By ethnicity, only 4.7% of the population is Hispanic or Latino while the rest are non-Hispanic (of any race). St. Louis is the most populous county (1,001,982 people), while Worth county is the least populated with only 2004 people (22). Although 87% (99/114) of the counties are classified as rural, only 33% of the population lives in rural areas (23).

### 2.3 Data sources

#### 2.3.1 COVID-19 data

Data on COVID-19 confirmed cases and fully vaccinated individuals reported from January 1 to December 31, 2021, were obtained from the Missouri Department of Health. A COVID-19 fully vaccinated individual was defined as a person who received either both doses of Pfizer-BioNTech or Moderna or one dose of the Johnson & Johnson vaccine. The data were aggregated to the county level and proportions of confirmed COVID-19 vaccinated population were computed using 5-year population estimates from the American Community Survey (ACS) as denominators (24).

#### 2.3.2 Sociodemographic and cartographic data

Data on sociodemographic factors such as age, gender, race and ethnicity, poverty, insurance, household size, educational attainment, employment, and commuting were obtained from the ACS 5-year estimates of 2016–2020 (24). These were investigated as potential predictors of proportion of county population vaccinated against COVID-19. Cartographic boundary files were downloaded from the US Census Bureau’s TIGER files and used for generating maps (25).

### 2.4 Descriptive analysis

Descriptive analysis was performed in GeoDa version 1.8 (26) and R version 4.1.1 (27) using the RStudio version 1.4.1717 (28) interface. The Shapiro–Wilk test was used to assess the normality of continuous variables. Non-normally distributed variables were summarized using median and the 1st and 3rd quartiles while mean and standard deviations were used for normally distributed variables (Table 1).

**Table 1:** Summary statistics of county-level predictors of proportions of COVID-19 vaccinated population in Missouri.

Type of variable	Variable	Median	1st quartile	3rd quartile
COVID-19 cases				
	Total cases	1,362	632	2,593
	Cases per 100 population	6.5	5.3	7.5
Demographic factors				
	% male population	49.5	49.0	50.4
	% white population	94.3	89.6	95.7
	% Black/African American population	1.2	0.5	3.9
	% Hispanic/Latino population	2.3	1.8	2.95
	% over 65	19.3	17.4	21.6
Economic variables				
	% below poverty level	15.4	12.4	18.1
	% uninsured population	11.3	8.4	14.0
	% unemployed population	2.5	2.0	3.1
	($) median household income	47,500.0	42,862.5	54,638.5
Educational variables				
	% with ≤ high school education	53.9	47.6	58.2
	% with some college education	20.7	19.1	23.0
	% with associate’s degree	7.8	6.8	8.8
	% with bachelor’s degree	11.8	9.1	14.7
Employment variables				
	% commute using public transportation	0.3	0.1	0.6
	% in agriculture^1^	4.4	2.5	7.1
	% in construction	7.5	5.9	9.3
	% in manufacturing	13.9	11.0	16.5
	% in retail trade	11.8	10.8	12.9
	% in transportation^2^	5.9	4.9	6.9
	% in education and health care^3^	22.3	20.6	26.2
	% in accommodation and food services^4^	6.6	5.3	8.7
Voting variables				
	% voted Republican in 2020 election	78.1	72.2	80.9

^1^Percent of population employed in agriculture, forestry, fishing, or hunting. ^2^Percent of population employed in transportation and warehousing and utilities. ^3^Percent of population employed in educational services and health care and social assistance. ^4^Percent of population employed in arts, entertainment, and recreation and accommodation and food services.

### 2.5 Investigation of predictors of county-level proportion of COVID-19 vaccinated population

#### 2.5.1 Global model

Univariable associations between each of the potential predictors and the log of the county-level proportion of COVID-19 vaccinated population were investigated using global Ordinary Least Squares (OLS) models (29) in GeoDa (26). A relaxed critical *p* -value of ≤0.15 was used to identify potentially significant predictors.

A multivariable global OLS model was then built in R using the manual backward elimination approach (*p* ≤ 0.05). Potential confounding variables were investigated using the change in parameter estimates method (30). Two-way interaction terms were investigated based on biological knowledge, and only the significant (*p* ≤ 0.05) ones were kept in the final mode. Simes method (31) was used to adjust for multiple testing. Collinearity among predictors of the final model was assessed using Multicollinearity Condition Number (MCN) in GeoDA and Variance Inflation Factor (VIF) in R. Adjusted R-squared (R^2^) and Akaike Information Criterion (AIC) were used to assess the overall goodness-of-fit.

#### 2.5.2 Local model

A Geographically Weighted Ordinary Least Squares (GWOLS) model was fit to the data to assess if the associations between county-level proportion of COVID-19 vaccinated population and each of the predictors changed based on geographical location. This local GWOLS model used the same outcome variable and predictors as the final global model. The local model was fit to the data in GWR version 4 (32) specifying an adaptive bi-square geographic kernel weight. The golden section search method was used to identify the optimum bandwidth. Model fit was assessed using small sample size bias-corrected Akaike Information Criteria (AICc). The geographic variability of each regression coefficient was investigated using the global OLS model’s Standard Error (SE), the local GWOLS model’s Interquartile Range (IQR), and the difference of criterion. Coefficients were considered non-stationary if IQR > 2*SE or the difference of criterion <−2 (33).

### 2.6 Cartographic display

All maps were generated in QGIS (34). Choropleth maps were used to display the geographic disparities of county-level proportion of COVID-19 vaccinated population, socioeconomic and demographic factors, and local regression coefficients of the GWOLS model. Critical intervals were determined using the Jenk’s optimization classification scheme.

## 3 Results

### 3.1 Descriptive statistics

A total of 1,362 (6.5%) of the population had confirmed COVID-19 during the study period. The majority (94.3%) were non-Hispanic White, while the median percentages of non-Hispanic Black and Hispanic/Latino populations across counties were 1.2 and 2.3%, respectively. About 19.3% were ≥ 65 years old, 2.5% were unemployed, and 11.3% did not have health insurance. Median household income was $47,500, with 15.4% living below the poverty level. Approximately 53.9% had high school education or less and 7.8% had an associate’s degree (Table 1). Regarding occupation, 22.3% worked in education and health care, 13.9% in manufacturing, and 11.8% in retail trade. The majority (78.1%) voted republican during the 2020 election.

### 3.2 Predictors of county-level proportion of COVID-19 vaccinated population

#### 3.2.1 Global model

The proportion of vaccinated population tended to be lower in counties with high percentages of the population that were Hispanic/Latino, uninsured, living below the poverty level, had high school education or less, worked in agriculture, worked in manufacturing, or voted Republican in the 2020 election (relaxed *p* = 0.15). On the other hand, the proportion of vaccinated individuals tended to be high in counties with high percentages of individuals that were public transport users, education and healthcare workers, had high median household income, bachelor’s degree, and COVID-19 cases (Table 2). No highly correlated variables were identified.

**Table 2:** Results of univariable and multivariable ordinary least squares regression models used to identify predictors of proportions of COVID-19 vaccinated population in Missouri.

Type of variable	Variable	Coefficient	95% confidence interval	*p*-values
Univariable model results				
COVID-19 cases	Total cases	9.978E-06	4E-06, 20E-06	<0.001
	Cases per 100 population	0.044	0.022, 0.066	<0.001
Demographic factors	% male population	−0.015	−0.037, 0.006	0.152
	% white population	0.001	−0.005, 0.006	0.777
	% Black/African American population	0.001	−0.005, 0.008	0.647
	% Hispanic/Latino population	−0.016	−0.031, −0.001	0.039
	% over 65	0.001	−0.009, 0.011	0.918
Economic variables	% below poverty level	−0.015	−0.023, −0.007	<0.001
	% uninsured population	−0.021	−0.031, −0.013	<0.001
	% unemployed population	−0.024	−0.069, 0.022	0.304
	($) median household income	6.712E-06	2E-06, 10E-06	<0.001
Educational variables	% with ≤ high school education	−0.008	−0.012, −0.004	<0.001
	% with some college education	0.004	−0.011, 0.019	0.612
	% with associate’s degree	0.007	−0.017, 0.031	0.552
	% with bachelor’s degree	0.016	0.007, 0.025	<0.001
Employment variables	% commute using public transportation	0.040	−0.012, 0.093	0.134
	% in agriculture^1^	−0.008	−0.018, 0.003	0.136
	% in construction	0.001	−0.015, 0.017	0.914
	% in manufacturing	−0.011	−0.019, 0.002	0.012
	% in retail trade	0.008	−0.010, 0.026	0.401
	% in transportation^2^	−0.014	−0.036, 0.009	0.232
	% in education and health care^3^	0.009	−0.001, 0.018	0.068
	% in accommodation and food services^4^	0.004	−0.010, 0.018	0.603
Voting variables	% voted Republican in 2020 election	−0.003	−0.007, 0.0004	0.079
Multivariable model results				
Demographic factors	% Hispanic/Latino population	−0.0153	−0.0304, −0.0002	0.046
Economic variables	% below poverty level	−0.0111	−0.0222, −0.0001	0.049
	% uninsured population	−0.0131	−0.0238, −0.0026	0.015

^1^Percent of population employed in agriculture, forestry, fishing and hunting. ^2^Percent of population employed in transportation and warehousing and utilities. ^3^Percent of population employed in educational services and health care and social assistance. ^4^Percent of population employed in arts, entertainment, and recreation and accommodation and food services.

Based on the final global multivariable model, low county-level proportions of COVID-19 vaccinated individuals tended to occur in counties with high percentages of Hispanic/Latino population (*p* = 0.046), individuals living below the poverty level (*p* = 0.049), and uninsured population (*p* = 0.015) (Table 2). The same three variables remained statistically significant in the final model after adjusting for multiple testing using Simes method since the corrected overall critical *p* -value was 0.05. Counties located in the eastern parts of the state tended to have high proportions of COVID-19 vaccinated population (Figure 1B) but low percentages of Hispanic/Latino (Figure 1C), uninsured (Figure 1D), and populations living below the poverty level (Figure 1E). Counties in the southernmost part of the state had the opposite distributions (Figures 1B,D,E).

#### 3.2.2 Local model

The regression coefficient for the association between proportion of COVID-19 vaccinated population and percentage of Hispanic/Latino population was non-stationary (IQR > 2*SE and difference of criterion <−2), implying that the strength of association changes across counties (Table 3). A west–east gradient was observed with strong negative associations being observed in counties of the westernmost and southcentral regions, while positive associations were observed in the eastern part of the state spanning from north to south (Figure 1F). There was no evidence of non-stationarity of the coefficients of percentages of uninsured or poor populations.

**Table 3:** Results of assessment of variability of the coefficients of the predictors of the proportions of COVID-19 vaccinated population in Missouri.

Name	Global SE^1^	Global SE^1^ x2	IQR^2^ of local coefficient	IQR^2^-2(SE)	Diff of criterion	Is coefficient varying?
% Hispanic/Latino population	0.0069	0.0137	0.0288	0.0151	−13.9602	Yes
% Below poverty level	0.0043	0.0086	0.0214	0.0128	5.2526	No^3^
% Uninsured population	0.0048	0.0096	0.0092	−0.0004	7.6352	No^4^

^1^Standard Error. ^2^Interquartile Range. ^3^Coefficients are non-varying based on Diff of Criterion. ^4^Coefficients are non-varying based on both IQR^2^-2(SE) and Diff of Criterion.

## 4 Discussion

This study investigated geographic disparities and predictors of county-level proportions of COVID-19 vaccinated population in Missouri from January to December 2021. The observed low proportions of COVID-19 vaccinated population in non-metropolitan communities in the southern part of the state might be due to inadequate healthcare facilities in these rural areas (23). Previous studies indicated that rural communities had higher burdens of diseases in general but lower access to healthcare resources than urban communities (35–39). According to a report by the Missouri Department of Health, counties in the southern part of the state tended to have fewer healthcare centers and primary care providers than the state average (40). Primary care providers play a crucial role in promoting vaccinations through dissemination of vaccine information as well as provision of vaccinations (29). Therefore, people living in these counties might have less access to vaccines due to lack of information on vaccine availability and access to vaccination centers. Additionally, the findings of this study identified that these counties had high percentages of uninsured individuals and those living below the poverty level, which could explain the low proportions of vaccinated population in these areas.

This study identified a significant negative association between county-level proportions of COVID-19 vaccinated population and the percentage of population living below the poverty level. These findings are consistent with those of previous studies, which reported that individuals with low income were less likely to get COVID-19 vaccines (41–44). This may be due to the fact that poor populations tend to have poor health literacy (45) and are unsure about the safety and effectiveness of the COVID-19 vaccine. Therefore, education on the safety and benefits of vaccines and addressing concerns about the vaccine side effects may help improve vaccination coverage among these populations.

This study found a significant association between the percentage of uninsured population and low proportions of COVID-19 vaccinated population which is consistent with reports by Donadio et al. that US counties with low health insurance coverage tended to have poor COVID-19 vaccination coverage (46). A study by Kelly et al. also reported that uninsured populations were 30% less likely to get COVID-19 vaccines than insured populations (7). Although COVID-19 vaccines are free to all, uninsured individuals may not know this due to lack of access to primary healthcare providers and fear of receiving bills. Vaccine hesitancy may be another reason for low vaccine uptake among uninsured populations. However, we acknowledge that vaccine hesitancy and low levels of vaccine uptake in populations are related but different; some individuals did not receive vaccines due to reasons other than vaccine hesitancy (47).

The significant negative association between county-level proportions of COVID-19 vaccinated population and percentages of Hispanic population suggests that race and ethnicity may play a role in vaccination disparities. A study by Khubchandani reported that COVID-19 vaccination hesitancy rates among Hispanic and African American adults were higher than the US average due to low education level, medical mistrust, and anti-vaccination beliefs (48). However, a study by Frisco et al. reported that US-born Hispanic adults were less vaccine hesitant compared to their White counterparts due to their experiences with COVID-19 (49). Since the Hispanic community in the US was greatly affected by COVID-19, they were more likely to have family members or friends who suffered or died from COVID-19. Such feelings motivated these populations to get vaccines. Suffice it to say that studies investigating vaccine hesitancy among Hispanic populations have produced mixed results (49). This could explain the non-stationary of associations between proportion of COVID-19 vaccinated population and percentage of Hispanic/Latino population across counties identified in this study.

### 4.1 Strengths and limitations

This is the first study investigating geographic disparities and predictors of proportions of COVID-19 vaccinated population at the county-level using global and local models in Missouri. In addition, this study identified how the associations varied across counties in Missouri. However, this study is not without limitations. Reporting of confirmed COVID-19 vaccination data could be inconsistent among counties and prone to reporting bias. Furthermore, this study investigated county-level geographic disparities and did not consider intra-county disparities. These limitations notwithstanding, the findings of this study provided useful information for guiding health planners in allocating healthcare resources and reducing disparities in COVID-vaccination in Missouri. Similar geographically weighted analysis could be used to investigate disparities of COVID-vaccination across states in the US.

## 5 Conclusion

The findings of this study confirm geographic disparities in COVID-19 vaccination in Missouri and suggest that certain socioeconomic conditions and race/ethnicity play significant roles in vaccination coverage. Therefore, study findings are useful for guiding education programs and resource allocation geared toward reducing disparities and promoting vaccinations in the state of Missouri.

## Data availability statement

The original contributions presented in the study are included in the article/Supplementary material, further inquiries can be directed to the corresponding author.

## Ethics statement

Ethical review and approval was not required for the current study in accordance with the local legislation and institutional requirements. Written informed consent for participation was not required for this study in accordance with the national legislation and the institutional requirements.

## Author contributions

AL: Conceptualization, Formal analysis, Investigation, Methodology, Validation, Visualization, Writing – original draft, Writing – review & editing. MK: Investigation, Visualization, Writing – original draft, Writing – review & editing. ND: Investigation, Visualization, Writing - Original draft, Writing - review & editing. PD: Conceptualization, Writing – review & editing. MI: Conceptualization, Investigation, Methodology, Writing – review & editing. SL: Conceptualization, Formal analysis, Investigation, Methodology, Supervision, Writing – original draft, Writing – review & editing. ALL: Conceptualization, Formal analysis, Investigation, Methodology, Validation, Writing – review & editing. CL: Conceptualization, Formal analysis, Funding acquisition, Investigation, Methodology, Project administration, Resources, Writing – original draft, Writing – review & editing. AO: Conceptualization, Formal analysis, Investigation, Methodology, Supervision, Validation, Visualization, Writing – original draft, Writing – review & editing.
